# Complex Inflammation mRNA-Related Response in ALS Is Region Dependent

**DOI:** 10.1155/2015/573784

**Published:** 2015-08-02

**Authors:** Sara Berjaoui, Mónica Povedano, Paula Garcia-Esparcia, Margarita Carmona, Ester Aso, Isidre Ferrer

**Affiliations:** ^1^Institute of Neuropathology, Bellvitge University Hospital, University of Barcelona, CIBERNED (Centro de Investigación Biomédica en Red de Enfermedades Neurodegenerativas), 08907 L'Hospitalet de Llobregat, Spain; ^2^ALS Unit, Service of Neurology, Bellvitge University Hospital, 08907 L'Hospitalet de Llobregat, Spain

## Abstract

Inflammatory changes are analyzed in the anterior spinal cord and frontal cortex area 8 in typical spinal-predominant ALS cases. Increased numbers of astrocytes and activated microglia are found in the anterior horn of the spinal cord and pyramidal tracts. Significant increased expression of* TLR7*,* CTSS*, and* CTSC* mRNA and a trend to increased expression of* IL10RA*,* TGFB1*, and* TGFB2* are found in the anterior lumbar spinal cord in ALS cases compared to control cases, whereas* C1QTNF7* and* TNFRSF1A* mRNA expression levels are significantly decreased.* IL6* is significantly upregulated and* IL1B* shows a nonsignificant increased expression in frontal cortex area 8 in ALS cases. IL-6 immunoreactivity is found in scattered monocyte-derived macrophages/microglia and TNF-*α* in a few cells of unknown origin in ALS cases. Increased expression and abnormal distribution of IL-1*β* occurred in motor neurons of the lumbar spinal cord in ALS. Strong IL-10 immunoreactivity colocalizes with TDP-43-positive inclusions in motor neurons in ALS cases. The present observations show a complex participation of cytokines and mediators of the inflammatory response in ALS consistent with increased proinflammatory cytokines and sequestration of anti-inflammatory IL-10 in affected neurons.

## 1. Introduction

Amyotrophic lateral sclerosis (ALS) is a progressive age-dependent neurodegenerative disease with an estimated incidence of about 1–3/100,000 resulting in death on average within 3–5 years of symptom onset. ALS is not a rare disease with lifetime risk of 1 : 1000 and 2 : 1 male predominance. Although ALS is primarily a motor neuron disease affecting the upper (cortical) and lower (brain stem and spinal) motor neurons with concomitant muscle atrophy, a proportion of patients develop frontotemporal lobar degeneration and occasionally other neurological symptoms. Therefore, ALS is a multisystem degeneration with predominant motor symptoms. About 8–10% of cases are inherited (fALS), the majority of them autosomal dominant but some of them recessive or X-linked. There is incomplete penetrance and intra- and interfamilial variability as well as variability at the age of onset and rate of progression. It has been suggested that about 13% of sALS cases bear a gene mutation linked to fALS [1, 2]. However, the cause of ALS in the majority of sporadic cases is not known.

Inflammation is a constant molecular phenomenon in ALS. This is manifested by a combination of altered systemic and central nervous system responses [3–9].

Regarding systemic responses, activated monocyte/macrophages [10], altered expression of chemokines in monocytes [11], increased levels of CD4+ cells, and decreased levels of CD8+ T lymphocytes [12] have been reported in ALS. Altered expression of several cytokines and mediators of inflammation has been reported in the serum although with remarkable differences from one laboratory to another, probably depending on the method used for determination. Increased protein expression of IL-17A [13, 14], IL-23 [14], IL-15 and IL-12 [14], chemokines [15], RANTES [16], IL-6 and TNF-*α* [17], TNF*α* and soluble receptors [18], and IL-6 [19] has been separately reported in the serum of ALS cases. However, only IL-18 and its endogenous inhibitor IL-18BP among several members of the IL-1 family were identified in a recent study [20].

Major inflammatory changes in the central nervous system in ALS involve increased numbers of reactive astrocytes and activated microglia in target regions such as the anterior horn of the spinal cord, motor nuclei of the brainstem, pyramidal tracts, and motor cortex, but also in other regions such as the prefrontal cortex and thalamus [21–25]. Increased inflammation has also been detected* in vivo* using the radioligand [11C]-(R)-PK11195, in motor cortex, dorsolateral prefrontal cortex, thalamus, and pons [26], and in tissue samples in ALS [27]. Intrinsic inflammatory-related cells in the spinal cord are accompanied by low numbers of lymphocytes and other blood-derived cells [28, 29].

Increased protein levels of several cytokines and mediators of the inflammatory response have also been reported in the CSF in ALS. These include IL-6 and IL-1*β* [30], RANTES [16], chemokines [15], IL-8 [31], IL-23 [32], IL-17A [13, 32], IL15 and IL-12 [14], chemokines [33], and IL-18BP [20]. This heterogeneous representation further indicates variations depending on the methods and products employed in the different laboratories.

Increased CSF levels of these molecules may be due to the active synthesis of inflammatory factors by intrinsic inflammatory cells, mainly microglia [34–37]. In this line, leukocyte common antigen (LCA), lymphocyte function associate molecule 1 (LFA-1), and complement receptors CR3 and CR4 are increased in the spinal cord [3, 23], together with cyclooxygenase 2 [38] and IFN*γ* and LIGHT [39] in ALS. Immunohistochemistry has also shown increased expression of TLR2, TLR4, and RAGE in reactive glial cells in both gray (ventral horn) and white matter of ALS spinal cord. TLR2 was predominantly detected in cells of the microglia/macrophage lineage, whereas the TLR4 and RAGE were strongly expressed in astrocytes [40].

Curiously, studies of mRNA expression are scant. High levels of mRNA and protein of classical complement pathway, C1q and C4, as well as the downstream complement components C3 and C5b-9, have been reported in ALS samples [36]. RT-qPCR analysis has confirmed the increased expression of both* TLR2* and* TLR4*, and* HMGB1* mRNA level in ALS patients paralleling increased TLR2 and TLR4 protein expression [40]. mRNA studies in the cerebral cortex are rarer, although upregulation of several cytokines and* IFNβ*, and encoding proteins involved in antigen presentation including major histocompatibility complex (MHC) class I molecules, has been reported in motor cortex [41].

The objective of the present study was to analyze with a large panel of mRNA probes the expression of cytokines and mediators of the immune response in the anterior lumbar spinal cord and frontal cortex area 8 in cases with sALS classical forms. The selection of probes was based on the fact that these are the same probes used to analyze inflammatory responses in other neurodegenerative diseases including Parkinson's disease, Alzheimer's disease, and Creutzfeldt-Jakob's disease [42–44]. This approach would permit the identification of disease-dependent commonalities and differences in the inflammatory responses examined by using the same probes in the same laboratory. The study was accompanied by immunohistochemical localization of selected inflammatory mediators in the anterior lumbar spinal cord. The immediate purpose was to assess inflammatory responses in two separate regions in ALS cases. The final goal was to gain understanding about the characteristics of intrinsic inflammation among different neurodegenerative diseases with abnormal protein aggregates to have basic information to provide a rationale for the specific use of eventual inflammation modulatory therapies in the different disorders.

## 2. Material and Methods

### 2.1. Brain and Spinal Cord Tissue Samples

Brain and spinal cord tissue samples were obtained from the Institute of Neuropathology Brain Bank (HUB-ICO-IDIBELL Biobank) following the Spanish legislation and after the approval of the local ethics committee. The postmortem delay ranged from 2 h 10 min to 16 h 30 min. After brain removal from the skull, one hemisphere was immediately cut in coronal sections, 1 cm thick, and selected areas of the encephalon were rapidly dissected, frozen on metal plates over dry ice, placed in individual air-tight plastic bags, numbered with water-resistant ink, and stored at −80°C until use. The other hemisphere was fixed by immersion in 4% buffered formalin for 3 weeks. Transversal sections of the cervical, thoracic, lumbar, and sacral spinal cortex were alternatively frozen at −80°C or fixed by immersion in 4% buffered formalin. Neuropathological examination in all cases was routinely performed on twenty selected dewaxed paraffin sections comprising different regions of the cerebral cortex, diencephalon, thalamus, brain stem, and cerebellum and several levels of the spinal cord, which were stained with haematoxylin and eosin, Klüver-Barrera, and, for immunohistochemistry to microglia, glial fibrillary acidic protein, *β*-amyloid, phosphorylated tau (clone AT8), *α*-synuclein, TDP-43, ubiquitin, and p62.

ALS cases (*n* = 14; mean age 64.4 years; 7 men and 7 women) had typical spinal-predominant clinical course and typical neuropathological findings of sporadic amyotrophic lateral sclerosis with TDP-43-immunoreactive intraneuronal inclusions [1]. Although the intensity of lesions in the pyramidal tracts and anterior spinal horn varied from one individual to another, cases were selected to minimize individual variations. Cases with cognitive impairment were not included in the present series. Neuropathological study also served to eliminate cases with combined pathologies including frontotemporal lobar degeneration associated with TDP-43pathy and cases with Alzheimer's disease- (AD-) related pathology at stages higher than I-II of Braak and Braak. Age-matched control cases (*n* = 19; mean age 64.8 years; 9 men and 10 women) had not suffered from neurologic, psychiatric, or metabolic diseases (including metabolic syndrome) and did not have abnormalities in the neuropathological examination excepting sporadic Alzheimer's disease- (AD-) related pathology stages I-II of Braak and Braak. ALS cases with more advanced stages of sporadic AD-related pathology were not considered in the present series to avoid overlap between ALS and AD in the frontal cortex. A summary of cases analyzed is shown in Table 1.

Biochemical studies were focused on the frontal cortex area 8 and anterior half of the lumbar spinal cord mainly comprising the anterior horns.

### 2.2. RNA Extraction and Purification

Extraction of RNA was performed with RNeasy Lipid Tissue Mini Kit (Qiagen, Hilden, DE) following the instructions provided by the supplier and performing the optional DNase digest to avoid extraction and later amplification of genomic DNA. The concentration of each sample was measured at 340 nm with the NanoDrop 2000 spectrophotometer (Thermo Scientific, Waltham, Massachusetts, USA). The RNA integrity number (RIN) was measured with the Agilent 2100 Bioanalyzer (Agilent, Santa Clara, California, USA), which is shown in Table 1.

### 2.3. Retrotranscription Reaction

The process was performed with the High-Capacity cDNA Archive kit (Applied Biosystems, Foster City, California, USA) according to the manufacturer's instructions and using Gene Amp 9700 PCR System thermocycler (Applied Biosystems). A negative control was used without reverse transcriptase to rule out any DNA contamination.

### 2.4. Real Time qPCR

RT-qPCR assays were done in duplicate on cDNA samples obtained from the retrotranscription reaction and were performed in 384-well optical plates (Applied Biosystems) utilizing the ABI Prism 7900 HT Sequence Detection System (Applied Biosystems). 20x TaqMan Gene Expression Assays and 2x TaqMan Universal PCR Master Mix (Applied Biosystems) were used for performing the amplification reactions. TaqMan probes used in this study are shown in Table 2. The selection of these probes was based on parallel studies using the same probes carried out in other neurodegenerative diseases, particularly AD, Parkinson's disease, and Creutzfeldt-Jakob's disease, for future comparative purposes. The reactions were performed following the sequence of temperature and time: 50°C for 2 min, 95°C for 10 min, 40 cycles at 95°C for 15 s, and 60°C for 1 min. TaqMan PCRs were recorded using the Sequence Detection Software (SDS version 2.3, Applied Biosystems). Threshold cycle (CT) data for each sample were analyzed. First, delta CT (ΔCT) values were calculated by normalizing the CT values of each target gene with the endogenous control *β*-glucuronidase (GUS-*β*) for normalization [45]. Second, ΔΔCT values were obtained with the ΔCT of each sample minus the mean ΔCT of the population of control samples (calibrator samples). The fold-change was determined using the equation 2^−ΔΔCT^.

### 2.5. Immunohistochemistry

Formalin fixed, paraffin embedded tissue sections 4-5 microns thick of anterior lumbar spinal cord from ALS cases and controls (*n* = 6 per group) were dewaxed and processed for CD68, IBA1, IL-6, IL-10, IL-1*β*, and TNF-*α* for immunohistochemistry. The sections were incubated with 2% hydrogen peroxide and 10% methanol for 30 min at room temperature, followed by 5% normal serum for 2 h. Then the sections were incubated overnight with one of the primary antibodies. Monoclonal mouse anti-human CD68 clone PG-M1CD68 antibody (Dako, Agilent Technologies, Barcelona, Spain) was used at a dilution of 1/50. IBA-1 rabbit, polyclonal antibody (Wako, Richmond, VA, USA) was used at a dilution of 1 : 250. Rabbit polyclonal antibodies against IL-6 (IL-6, ab6672, Abcam) were diluted 1/100, IL-10 (AP52181PU-N, Acris) 1/1,000; IL-1*β* (IL1 beta, ab9722, Abcam) 1/200; mouse monoclonal antibodies against TNF-*α* (ab1793, Abcam) were diluted 1/10. Peroxidase reaction was visualized with diaminobenzidine and H_2_O_2_. Control of the immunostaining included omission of the primary antibody; no signal was obtained following incubation with only the secondary antibody.

### 2.6. Double-Labelling Immunofluorescence and Confocal Microscopy

Double-labeling immunofluorescence was carried out on dewaxed sections, 4 microns thick, which were stained with a saturated solution of Sudan black B (Merck, DE) for 15 min to block the autofluorescence of lipofuscin granules present in cell bodies and then rinsed in 70% ethanol and washed in distilled water. Antigenicity enhancement was performed by boiling the sections in citrate buffer. The sections were next blocked for 30 min at room temperature with 10% fetal bovine serum diluted in PBS. Then, the sections were incubated at 4°C overnight with rabbit polyclonal anti-IL-10 (AP52181PU-N, ACRIS) and anti-phospho TDP-43 mouse monoclonal antibody (pS409/410-1; Cosmo Bio Co, Japan). After washing, the sections were incubated with Alexa488 or Alexa546 (1 : 400, Molecular Probes, USA) fluorescence secondary antibodies against the corresponding host species. Nuclei were stained with DRAQ5 (1 : 2,000, BioStatus, GB). After washing, the sections were mounted in Immuno-Fluore mounting medium (ICN Biomedicals, USA), sealed, and dried overnight. Sections were examined with a Leica TCS-SL confocal microscope.

### 2.7. Gel Electrophoresis and Western Blotting

Samples of the spinal cord were homogenized in RIPA lysis buffer (50 mM Tris/HCl buffer, pH 7.4 containing 2 mM EDTA, 0.2% Nonidet P-40, 1 mM PMSF, protease, and phosphatase inhibitor cocktails, Roche Molecular Systems, USA). The homogenates were centrifuged for 15 min at 13,000 rpm. Protein concentration was determined with the BCA method (Thermo Scientific). Equal amounts of protein (20 *μ*g) for each sample were loaded and separated by electrophoresis on sodium dodecyl sulfate polyacrylamide gel electrophoresis (SDS-PAGE) (10%) gels and transferred onto nitrocellulose membranes (Amersham, Freiburg, Germany). Nonspecific bindings were blocked by incubation in 3% albumin in PBS containing 0.2% Tween for 1 h at room temperature. After washing, membranes were incubated overnight at 4°C with the antibodies against IL-1*β* (1 : 5,000, Abcam, Cambridge, UK), IL-6 (1 : 1,000, Abcam), and cathepsin S (1 : 500, Santa Cruz Biotechnology, Dallas, TX, USA). Protein loading was monitored using an antibody against *β*-actin (1 : 30,000, Sigma-Aldrich, St. Louis, MO, USA). Membranes were then incubated for 1 h in the appropriate HRP-conjugated secondary antibodies (1 : 2,000, Dako, Glostrup, Denmark), and immunocomplexes were revealed by chemiluminescence reagent (ECL, Amersham GE Healthcare, Buckinghamshire, UK). Densitometric quantification was carried out with TotalLab version 2.01 software (Pharmacia, Sweden). Bands were normalized to *β*-actin. Thirteen ALS samples and twelve control samples were analyzed.

### 2.8. Statistical Analysis

The normality of distribution of the mean fold-change values obtained by RT-qPCR was analyzed with the Kolmogorov-Smirnov test. Results were analysed with Student's *t*-test. Differences between groups were considered statistically significant at ^*∗*^ *p* < 0.05 and ^*∗∗*^ *p* < 0.01.

## 3. Results

### 3.1. mRNA Expression Assessment by RT-q PCR

17 mRNAs were selected in this study and their expression was tested in the frontal cortex (13 ALS cases and 14 control cases) and the anterior half of the lumbar spinal cord (12 ALS cases and 9 control cases). The study included members of the complement system (*C1QTNF7*,* C3AR1*), colony stimulating factors (*CSF3R*), Toll family (*TLR4, TLR7*), cytokines (*IL6, IL6ST, IL1B*),* TNFα* family (*TNFRSF1A*,* TNF-α*),* IL10* (*IL10*,* IL10RA*),* TGFβ* family (*TGFβ1*,* TGFβ2*), and cathepsins (*CTSS*,* CTSC*).* GUS-β* was used for normalization. Some samples from control and ALS patients had also AD-related pathology at stages I-II of Braak and Braak. In order to elucidate whether first stages of AD-related pathology might affect the inflammatory process in the spinal cord and therefore distort the specific inflammatory response related to ALS, a preliminary AD versus non-AD comparison in control and ALS samples of the spinal cord was performed. No differences due to AD-related pathology were observed in the spinal cord between control and ALS cases (Supplementary Table  I in Supplementary Material available online at http://dx.doi.org/10.1155/2015/573784).

#### 3.1.1. Anterior Lumbar Spinal Cord

*TLR7*,* CTSS*,* and CTSC* mRNA significant upregulation (*p* < 0.05) was found in the anterior lumbar spinal cord in ALS cases compared to control cases.* IL10RA*,* TGFB1*, and* TGFB2* showed a tendency to increase without statistical significance.* C1QTNF7* and* TNFRSF1A* mRNA were downregulated (*p* < 0.05). No modification was found in the expression of the rest of the genes studied in this region (Table 3).

#### 3.1.2. Frontal Cortex Area 8

Since previous studies have shown early modifications in mRNA cytokine expression in the frontal cortex at early stages of AD-related pathology [44], studies of mRNA in this region were limited to control and ALS without AD-related pathology. The number of remaining cases (ALS = 9; control = 9) was considered sufficient for comparative analysis.

*IL6* was significantly upregulated (*p* < 0.05) and* IL1B* showed a nonsignificant increase in ALS cases when compared to control cases (Table 3).

### 3.2. Protein Quantification by Western Blotting

**Figure 1:**
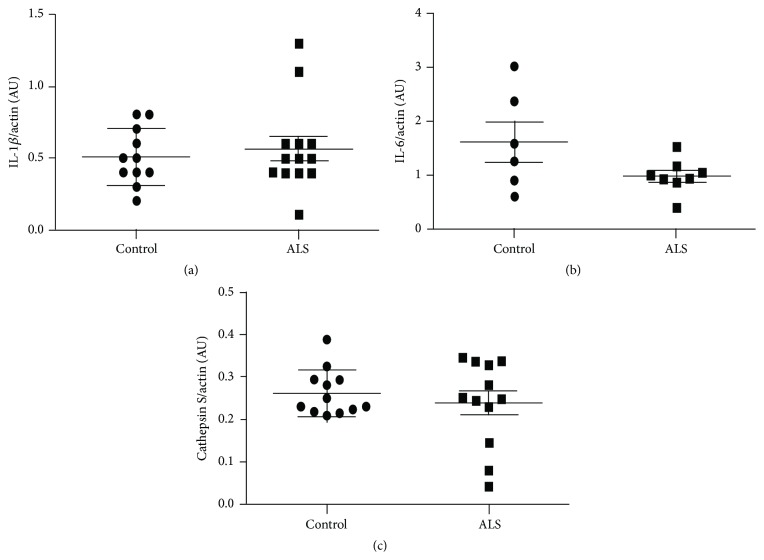
Quantification of western blotting of IL1*β* (a), IL-6 (b), and cathepsin s (c) in lumbar spinal cord shows no significant differences in control and ALS cases. *β*-actin levels were used as loading control. Densitometric quantifications are expressed as mean values ± SD.

The total levels of three of proteins were quantified by western blotting. No significant difference was observed between control and ALS regarding IL-1*β*, IL-6, and cathepsin S protein levels (Figures 1(a)–1(c)).

### 3.3. Immunohistochemistry, Double-Labelling Immunofluorescence, and Confocal Microscopy

**Figure 2:**
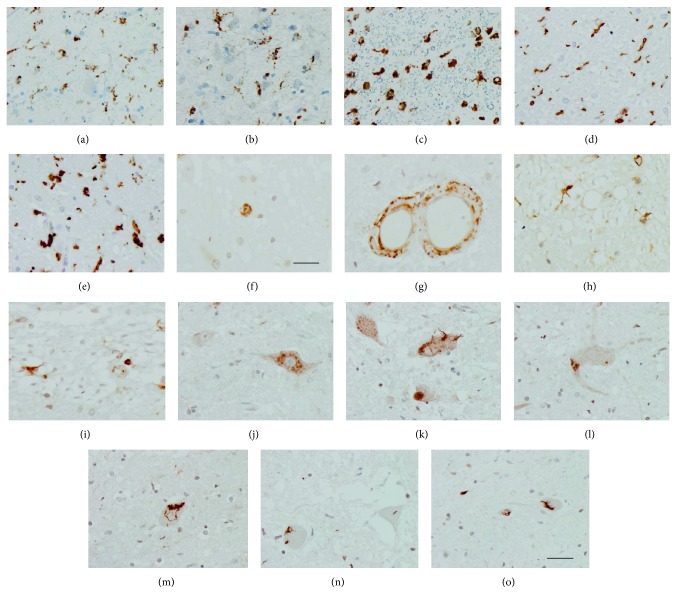
(a–c): CD68 immunohistochemistry shows amoeboid monocyte derived macrophages/microglia in the anterior lumbar horn (a and b) and pyramidal tracts (c) of the lumbar spinal cord in control (a) and ALS (b and c). (d and e) IBA1 stains immunoreactivity is present in large numbers of monocyte derived macrophages/microglia in the lateral pyramidal tract in control (D) and ALS cases with predominance of amoeboid cells in disease. (f) TNF-*α* is localized in a few round cells of undetermined origin. (g–i) IL-6 immunoreactivity is found in the wall of blood vessels (g) and in scattered microglial cells (h and i). (j and k) IL-1*β* immunoreactivity is present in neurons in control (j) and ALS cases but increased immunoreactivity and abnormal distribution of IL-1*β* is observed in a few motor neurons of the lumbar spinal cord only in ALS (k). (l–o) Strong IL-10 immunoreactivity is seen in cytoplasmic inclusions in ALS reminiscent of ubiquitinated, TDP-43-positive inclusions currently found in motor neurons in sALS. Paraffin sections, slight haematoxylin counterstaining, bar in (o) valid for all figures = 30 *µ*m, excepting bar in* F* = 50 *µ*m.

CD68 immunohistochemistry revealed that the phenotype of monocyte derived macrophages/microglia was ramified and amoeboid in the anterior lumbar horn, anterior root, and direct and crossed pyramidal tracts of the spinal cord in ALS (Figures 1(a)–1(c)). Differences in number were assessed regarding IBA1-immunoreactive cells; increased numbers of monocyte derived macrophages/microglia (543.1 ± 36.2 cells/mm^2^ in ALS versus 312.6 ± 19.3 cells/mm^2^ in controls), in addition to high predominance of amoeboid cells, were found in the pyramidal tracts of the spinal cord in ALS (Figures 2(d) and 2(e)). Yet TNF-*α* was restricted to a few round cells of unknown origin (Figure 2(f)). Strong IL-6 immunoreactivity occurred in the wall of the blood vessels (Figure 2(g)) and in scattered glial cells with the morphology of microglia/macrophage in ALS (Figures 2(g) and 2(h)). Increased IL-1*β* immunoreactivity was observed in motor neurons in ALS (118.7 ± 24.5 cells/mm^2^) when compared to controls (64.3 ± 15.0 cells/mm^2^); abnormal distribution of IL-1*β* was also found in a few motor neurons of the lumbar spinal cord only in ALS (Figures 2(i) and 2(j)). IL-10 immunoreactivity was present in neurons in control (41.5 ± 4.3 cells/mm^2^) and ALS cases (63.5 ± 20.2 cells/mm^2^). Curiously, increased IL-10 immunoreactivity was seen in cytoplasmic inclusions reminiscent of ubiquitinated, TDP-43-positive inclusions currently seen in motor neurons in sALS (Figures 2(k)–2(o)).

**Figure 3:**
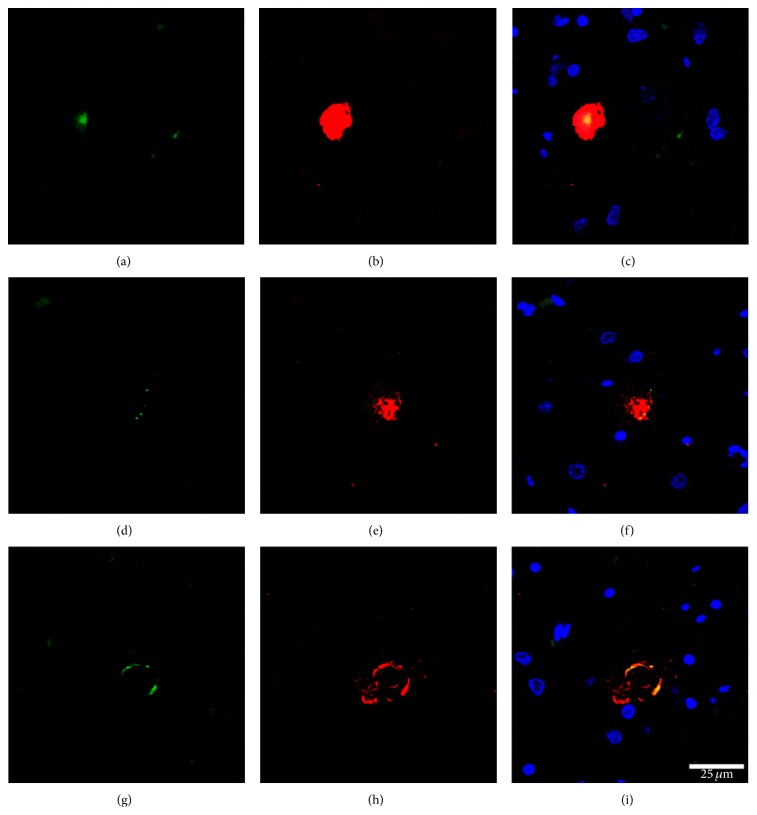
Double-labelling immunofluorescence and confocal microscopy show partial colocalization of IL-10 (green, a, d, g) and phosphorylated TDP-43 Ser409-410 (red, b, e, h) in globular and skein-like inclusions in motor neurons in ALS cases; merge (c, f, i); nuclei (blue) are with DRAQ5; bar = 25 *µ*m.

Double-labelling immunofluorescence and confocal microscopy further supported colocalization of IL-10 and phosphorylated TDP-43 Ser409-410 in globular and skein-like inclusions of motor neurons in ALS, involving about 80% of TDP-43-immunoreactive inclusions (Figure 3). No increased IL-10 immunoreactivity has been seen in glial cells containing phosphorylated TDP-43 Ser409-410 inclusions.

## 4. Discussion

The present findings demonstrate intrinsic regulation of cytokines and mediators of the immune response in the anterior part (roughly anterior horn) of the lumbar spinal cord and frontal cortex area 8 in ALS cases compared to age-matched controls. Significant increased expression of* TLR7*,* CTSS*, and* CTSC* mRNA and a trend to increased expression of* IL10RA*,* TGFB1,* and* TGFB2* were found in the anterior lumbar spinal cord in ALS cases compared to control cases, whereas* C1QTNF7* and* TNFRSF1A* mRNA expression levels are significantly decreased.* IL6* is significantly upregulated and* IL1B* shows a nonsignificant increased expression in frontal cortex area 8 in ALS cases when compared to control cases. The analysis of the frontal cortex area 8 is considered pertinent as it is vulnerable to ALS and major target of cases with associated frontotemporal lobar degeneration (FTLD) [1]. The present findings further show upregulation of key cytokines even in cases with no concomitant FTLD.

These results indicate regional differences in the intrinsic inflammatory response in ALS with higher deregulation in the anterior lumbar spinal cord in comparison to the frontal cortex area 8. Interestingly, not the same mediators were equally deregulated in the spinal cord and frontal cortex.

No relation between altered mRNA expression and total protein contents of IL-1*β*, IL-6, and cathepsin S is found in ALS cases. Incomplete correlation between mRNA and protein values can be explained by the modulation of mRNA translation by several species of noncoding RNAs. Protein expression of regulated cytokines by microRNAs has been reported in ALS [46]. However immunohistochemistry has shown increased IL-6 immunoreactivity in scattered cells with monocyte derived macrophages/microglia morphology. Altered IL-1*β* immunoreactivity, consistent with cytoplasmic aggregates, occurred in a few motor neurons in ALS. These findings together with monocyte derived macrophages/microglia morphology and increased TNF-*α* immunoreactivity in a few mononuclear cells of unknown origin in the anterior horn in ALS support the idea that monocyte derived macrophages/microglia at end-stages of ALS have cytotoxic properties [25, 34, 35, 37]. Increased IL-1*β* and IL-6 protein levels have also been detected in the CSF in ALS [30], thus suggesting secretion of these factors from the nervous system to the CSF.

IL-6 has anti-inflammatory and proinflammatory effects [47, 48]. IL-6 binds to the IL-6 receptor which, in turn, binds to the protein gp130, thus producing a restricted response in the presence of IL-6 receptor; this is the classic signaling pathway modulating anti-inflammatory responses. However, only a few cells express the IL-6 receptor whereas all cells bear gp130 protein on the cell surface. Direct activation of IL-6 of gp130 produces an extended response with damaging consequences [49, 50]. Transsignaling is a dominant mechanism for the pathogenic actions of interleukin 6 in the brain [51].

IL-1*β* is a proinflammatory cytokine that can produce structural damage to neurons and neuronal dysfunction by acting on glutamate-mediated excitatory postsynaptic currents [52–54]. Chronic administration of IL-1*β* leads to neurodegeneration [55]. Chronic increased expression of both cytokines may produce a harmful effect on motor neurons in the ALS context.

Previous studies have shown that deregulation of TDP-43 and p65 subunits triggers NF*κ*B-mediated pathogenic pathways in the spinal cord of ALS cases and that TDP-43 also increases the production of proinflammatory cytokines in microglia under appropriate settings [56]. An unexpected finding in the present study was the colocalization of IL-10 and TDP-43-positive cytoplasmic inclusions in anterior horn motor neurons in ALS. TDP-43 participates in IL-6 and IL-10 processing at the level of the subnuclear body called the interleukin 6 and 10 splicing activating compartment which is a nuclear site of cytokine RNA production and stability [57]. But to our knowledge no TDP-43 and IL-10 protein interactions have ever been reported. IL-10 is a potent anti-inflammatory cytokine that regulates various anti-inflammatory pathways [58–60]. However, the present findings show that IL-10 is sequestered at the TDP-43-immunoreactive inclusions in ALS motor neurons, thus suggesting loss of its potential beneficial function.

Neuroinflammation has also been examined using the same probes in several regions and stages of disease progression in Alzheimer's disease, Parkinson's disease, and Creutzfeldt-Jakob's disease [42–44]. Those studies have shown region- and stage-dependent differences in the inflammatory responses but also disease-dependent inflammatory profiles. Moreover, these patterns differ from those identified here in the frontal cortex area 8 and anterior lumbar spinal cord in ALS at terminal stages. Therefore, inflammation-related responses are not identical and homogeneous through different neurodegenerative diseases with abnormal protein aggregates, but rather they manifest specific patterns. The patterns also vary with disease progression in human diseases and related animal models [43, 44]. Since only ALS at terminal stages was explored in the present study, there is no information regarding inflammatory changes with disease progression in human ALS cases.

Inflammation in ALS is a very complex phenomenon [5, 8, 9]. Previous clinical trials using unique anti-inflammatory compounds have produced limited benefits [61–63] with the exception of Tocilizumab, an inhibitor of IL-6 receptor that has been shown to decrease inflammation somewhat in ALS [64, 65]. However, IL-6 is not the only cytokine involved in ALS, and combined therapies are probably needed to cope with the multiple pathways and variegated molecules that converge in the process of inflammation in ALS.

## Supplementary Material

No significant differences between mRNA expression in the anterior lumbar spinal cord was observed between samples from patients diagnosed or not for AD either in control or ALS groups. Values are calculated with the ΔΔCT method, using GUS-B as housekeeping gene and control samples as references. N.S.: Not significant.

## Figures and Tables

**Table 1:** Summary of clinical and pathological data in the present series.

Case	Pathology	Sex	Age	P-M	RIN FC	RIN SC
1	0	M	43	5 h 55 m	8.1	6.5
2	0	M	47	4 h 55 m	7.8	
3	0	M	46	15 h	7.7	6
4	0	F	71	8 h 30 m	7.4	6
5	0	M	52	3 h	8.2	
6	0	M	64	8 h 30 m	7.7	
7	0	M	67	5 h	7.4	
8	0	F	49	7 h	8.2	
9	0	F	75	3 h	7.7	
10	0	F	64	5 h		7.1
11	AD II	F	86	4 h 15 m		
12	AD I	F	79	3 h 35 m		
13	AD I	F	79	6 h 25 m		6.9
14	AD II	F	77	3 h 15 m		
15	AD II	F	76	5 h 45 m		
16	AD I	F	59	11 h 20 m		6.9
17	AD I	M	76	6 h 30 m		6.8
18	AD I	M	56	7 h 10 m		6.3
19	AD II	M	66	4 h 55 m		6.4
20	ALS/ADI	F	76	13 h		6.6
21	ALS	F	59	14 h 15 m	6.5	
22	ALS	M	54	4 h 50 m	7.7	
23	ALS	M	70	3 h	7.2	7.2
24	ALS	M	56	10 h 50 m		7.4
25	ALS	M	77	4 h 30 m	8	7.9
26	ALS/AD II	F	75	4 h 5 m		7
27	ALS	F	57	10 h	7.4	6.7
28	ALS	F	56	3 h 45 m	8.3	8.3
29	ALS/AD II	M	57	4 h		6.5
30	ALS/AD II	F	79	2 h 10 m		7.4
31	ALS	M	64	16 h 30 m	7.4	7
32	ALS	M	59	3 h 15 m	7.8	7.5
33	ALS	F	63	13 h 50 m	7.4	6.9

Thirty-three cases are analyzed corresponding to 19 controls and 14 amyotrophic lateral sclerosis (ALS) cases. Some ALS cases have concomitant AD-related pathology at stages I-II of Braak (AD I-II), and for this reason some control cases with similar stages of AD-related pathology are included in the study of the anterior lumbar spinal cord. However, only cases without AD-related pathology are selected for study in the frontal cortex area 8 (see text). M: male; F: female; P-M: postmortem delay (hours, minutes); FC: frontal cortex area 8; SC: anterior lumbar spinal cord; RIN: RNA integrity number.

**Table 2:** Abbreviation of genes examined and full names and sequences of TaqMan probes used in the present study.

Gene	Full name	Sequence of TaqMan probes
*GUS-β*	*β*-glucuronidase	GCTACTACTTGAAGATGGTGATCGC
*C1QTNF7 *	C1q and tumor necrosis factor related protein 7	GGGAACTGCAGGTTTGAGAGGTAAG
*C3AR1 *	Complement component 3a receptor 1	TCTCAGTTTTTTGAAGTTTAGCAAT
*CSF3R *	Colony stimulating factor 3 receptor	GCTGCTCCCCGGAAGTCTGGAGGAG
*CTSC *	Cathepsin C	CGGTTATGGGACCACAAGAAAAAAA
*CTSS *	Cathepsin S	AAAGCCATGGATCAGAAATGTCAAT
*IL1B *	Interleukin 1*β*	CAGATGAAGTGCTCCTTCCAGGACC
*IL6 *	Interleukin 6	TCAGCCCTGAGAAAGGAGACATGTA
*IL6ST *	Interleukin 6 signal transducer	CAAAGTTTGCTCAAGGAGAAATTGA
*IL8 *	Interleukin 8	GTGTGAAGGTGCAGTTTTGCCCAAGG
*IL10 *	Interleukin 10	AATAAGCTCCAAGAGAAAGGCATCT
*IL10RA *	Interleukin 10 receptor *α*	CAGTGTCCTGCTCTTCAAGAAGCCC
*TGFB1 *	Transforming growth factor *β*1	AGTACAGCAAGGTCCTGGCCCTGTA
*TGFB2 *	Transforming growth factor *β*2	GCACAGCAGGGTCCTGAGCTTATAT
*TLR4 *	Toll-like receptor 4	GGAGCCCTGCGTGGAGGTGGTTCCT
*TLR7 *	Toll-like receptor 7	AGACTAAAAATGGTGTTTCCAATGT
*TNF-α*	Tumor necrosis factor *α*	TGGCCCAGGCAGTCAGATCATCTTC
*TNFRSF1A *	Tumor necrosis factor receptor superfamily, member 1A	CTCCTGTAGTAACTGTAAGAAAAGC

**Table 3:** Results of mRNA expression assessed by RT-qPCR using GUS-*β* for normalization.

	Frontal cortex			Spinal cord		
	Control	ALS	*p* value	Control	ALS	*p* value
*Proinflammatory signaling *						
Complement system						
C1QTNF7	1.14 ± 0.63	1.42 ± 0.89		1.07 ± 0.43	0.52 ± 0.41	*∗*
C3AR1	1.23 ± 0.66	1.53 ± 0.94		1.00 ± 0.10	0.94 ± 0.41	
Colony stimulating factor						
CSF3R	1.16 ± 0.59	1.38 ± 0.69		1.07 ± 0.45	1.37 ± 0.88	
Toll-like receptor						
TLR4	1.29 ± 0.85	0.93 ± 0.34		1.26 ± 0.91	0.95 ± 0.19	
TLR7	1.16 ± 0.59	1.05 ± 0.50		1.08 ± 0.39	2.12 ± 0.90	*∗*
Cathepsins						
CTSC	1.15 ± 0.64	1.62 ± 0.91		1.11 ± 0.52	1.71 ± 0.67	*∗*
CTSS	1.26 ± 0.83	1.36 ± 0.74		1.05 ± 0.35	2.01 ± 0.93	*∗∗*
Interleukins						
IL1B	1.32 ± 0.89	3.29 ± 2.70	0.053	1.27 ± 0.81	1.13 ± 1.05	
IL6	1.42 ± 1.29	5.11 ± 4.73	*∗*	1.69 ± 2.19	3.04 ± 2.97	
IL6ST	1.04 ± 0.33	1.23 ± 0.48		1.05 ± 0.35	0.84 ± 0.17	
IL8	2.19 ± 3.91	1.06 ± 1.16		1.46 ± 1.43	1.41 ± 1.26	
TNF family						
TNF-*α*	ND	ND		1.11 ± 0.57	0.95 ± 0.31	
TNFRSF1A	1.10 ± 0.52	1.41 ± 0.86		1.04 ± 0.30	0.71 ± 0.11	*∗*

*Anti-inflammatory signaling *						
IL-10 family						
IL10	1.17 ± 0.58	1.40 ± 0.68		1.20 ± 0.70	1.54 ± 0.61	
IL10RA	1.13 ± 0.56	1.27 ± 0.75		1.07 ± 0.39	1.55 ± 0.71	0.087
TGF beta family						
TGFB1	1.08 ± 0.45	1.19 ± 0.54		1.01 ± 0.15	1.73 ± 0.85	0.063
TGFB2	1.18 ± 0.68	1.41 ± 0.69		1.01 ± 0.17	1.64 ± 0.90	0.07

*TLR7*, *CTSS,* and *CTSC* mRNA are significantly upregulated, and *C1QTNF7* and TNFRSF1A mRNA are significantly downregulated in the anterior lumbar spinal cord in ALS; a tendency to increase is also observed for *IL10RA*, *TGFB1,* and *TGFB2*. *IL6* is significantly upregulated and *IL1B* shows a trend to increase in the frontal cortex area 8 in ALS compared to controls. Values are calculated with the ΔΔCT method, using *GUS-B* as housekeeping gene and control samples as references. ND: not detectable. ^*∗*^ *p* < 0.05; ^*∗∗*^ *p* < 0.01 versus controls.
